# Rapid and robust patterns of spontaneous locomotor deficits in mouse models of Huntington’s disease

**DOI:** 10.1371/journal.pone.0243052

**Published:** 2020-12-28

**Authors:** Taneli Heikkinen, Timo Bragge, Niina Bhattarai, Teija Parkkari, Jukka Puoliväli, Outi Kontkanen, Patrick Sweeney, Larry C. Park, Ignacio Munoz-Sanjuan

**Affiliations:** 1 Charles River Discovery Services, Kuopio, Finland; 2 School of Pharmacy, Faculty of Health Sciences, University of Eastern Finland, Kuopio, Finland; 3 Naason Science Inc., Chungcheongbuk-do, South Korea; 4 CHDI Management/CHDI Foundation, Los Angeles, California, United States of America; University of Florida, UNITED STATES

## Abstract

Huntington's disease (HD) is an inherited neurodegenerative disorder characterized by severe disruption of cognitive and motor functions, including changes in posture and gait. A number of HD mouse models have been engineered that display behavioral and neuropathological features of the disease, but gait alterations in these models are poorly characterized. Sensitive high-throughput tests of fine motor function and gait in mice might be informative in evaluating disease-modifying interventions. Here, we describe a hypothesis-free workflow that determines progressively changing locomotor patterns across 79 parameters in the R6/2 and Q175 mouse models of HD. R6/2 mice (120 CAG repeats) showed motor disturbances as early as at 4 weeks of age. Similar disturbances were observed in homozygous and heterozygous Q175 KI mice at 3 and 6 months of age, respectively. Interestingly, only the R6/2 mice developed forelimb ataxia. The principal components of the behavioral phenotypes produced two phenotypic scores of progressive postural instability based on kinematic parameters and trajectory waveform data, which were shared by both HD models. This approach adds to the available HD mouse model research toolbox and has a potential to facilitate the development of therapeutics for HD and other debilitating movement disorders with high unmet medical need.

## Introduction

Mammalian locomotion is composed of a sequence of musculoskeletal positions to exert force **[1,2]** and, when compromised, organisms can adapt and uncouple these components to prioritize efficiency **[3–5]**. These adaptations for the economy of movement can be measured as changes in gait. In patients, the degeneration of critical brain regions for movement or posture control, e.g., basal ganglia, leads to progressive alterations in gait **[6–10]**. Neuroprotection for such disorders is likely to be most successful when targeted to the early phases of neural dysfunction before significant neuronal loss, placing an emphasis on the development of disease models that demonstrate early and progressive phenotypes in movement and gait. Such phenotypes have been observed in genetic, toxin-induced and surgical models of neurological disorders **[11–17]**. However, measuring quantitative changes in the complex adaptive movement of aging diseased mice is challenging.

Huntington's disease (HD) is a progressive neurodegenerative disorder caused by an expansion of the CAG trinucleotide repeat region in the huntingtin (*HTT*) gene on chromosome 4 **[18]**. Although HD is characterized by multiple movement, psychiatric and cognitive disturbances, its diagnosis is ultimately confirmed on the basis of the positive genetic test and onset of motor symptoms as measured by the Unified HD Rating Scale (UHDRS) total motor score (TMS) **[19,20]**. UHDRS-TMS comprises several criteria, including the presence of bradykinesia and altered gait pattern **[21]**. Bradykinesia—expressed as decreased velocity and stride length—can be detected in HD gene-expansion carriers (HDGECs) already in the presymptomatic phase **[22,23]**. As disease progresses, these manifestations worsen in HDGECs, and their cadence, joint angle range and stride length decrease further **[24,25]**. Significantly increased intra-individual variability of gait parameters such as stride length, stance time and swing time are typical features of HD **[25–27]**.

Multiple genetically engineered mouse models expressing mutated *HTT* have been developed that show aspects of HD-like pathology and neurological symptoms. These mouse lines can be divided broadly into three types based on the mutant *HTT* (m*HTT*) construct: transgenic models expressing either N-terminal fragments of the m*HTT* gene or full-length m*HTT*, and knock-in (KI) models, in which CAG repeats are inserted into the endogenous mouse *Htt* gene (for review, see **[28]**). Neurological symptoms have been most extensively studied in HD mouse models using tests such as rotarod, open field, grip strength and footprint test for the evaluation of gross cadence changes **[29]**. Alterations in several gait parameters in these mouse models could be correlated to the observations in patients. For example, shorter stride length was noted in 24-month-old *Hdh*^*Q111*^ KI heterozygous and homozygous mice and 12-month-old homozygous *Hdh*^*(CAG140)*^ mice **[30,31]**. The mean stride and base lengths did not differ in 12-month-old heterozygous *Hdh*^(CAG)150^ KI mice and wild-type counterparts but the intra-individual variability of these indices was considerably higher in the mutants **[32]**. Being potentially useful for phenotypic and pharmacological screening, the footprint test nonetheless does not characterize the full spectrum of gait deficits of HD mouse models. Moreover, in some widely used HD lines, gait changes revealed by the footprint test are minor and transient (e.g., as in BACHD mice **[29]**) and/or appear very late in life (13.5 months in YAC128 line **[33]** and 24 months in the *Hdh*^*Q111*^ KI line **[30]**).

To provide a more robust method of assessing locomotor behavior relevant to HD symptoms, we have developed a novel kinematic analysis approach based on our previous work **[13]** and tested it in two popular HD models, R6/2 and Q175. R6/2 mice express exon 1 of m*HTT* carrying a 141–157 CAG repeat, which triggers rapid progression of the pathology: the mutant mice start to lose body weight and develop motor and cognitive deficits at 6–8 weeks of age and die early **[34]**. *Q175 mice have the human exon 1 sequence with approximately 190 CAG repeats knocked into the endogenous Htt gene*; they show age- and genotype-dependent progression of motor, cognitive, and electrophysiological deficits, accompanied by decreased brain volumes and striatal metabolic changes, which develop more slowly than in the R6/2 mice **[35,36]**. Motor changes in Q175 mice typically present as hypoactivity in homozygotes from 2 months and in heterozygotes from 4 months of age **[36]**. We selected R6/2 and Q175 mice to test our new approach for the following reasons. R6/2 mice are by far the most popular HD mouse model with a clear gait phenotype revealed by different tests **[13,29,37,38]**, therefore it could be a suitable benchmark. In contrast to the situation with R6/2 mice, motor symptoms in Q175 KI mice progress over several months, particularly in heterozygous animals, and their gait has not been analyzed in detail previously. Judging from at best moderate gait phenotypes in other KI HD mouse models **[29–32]**, Q175 mice would provide a good sensitivity test of our method. In our high precision kinematic analysis, we measured 79 parameters of gait, body posture and fine motor movements during early, middle and advanced phases of the disease in R6/2 and Q175 KI mice of both sexes. Five principal components of mouse movements were identified that captured main features of mouse gait. In addition, two generalized phenotypic scores based on kinematic parameters and trajectory waveform patterns, respectively, have been derived. We expect that these indices can be more broadly applied to mouse models of other movement disorders to determine treatment effects and potentially be utilized in neurotoxicology applications.

## Materials and methods

### Animals

Experiments were conducted at Charles River Discovery Services, Kuopio, Finland according to the National Institute of Health (NIH) Guidelines for the Care and Use of Laboratory Animals and approved by the State Provincial Office of Southern Finland. R6/2 and respective wild-type (WT) littermates (120 CAGs) were obtained from the Jackson Laboratory (JAX Stock No: 006494, The Jackson Laboratory, Bar Harbor, ME, USA) **[34]**. Heterozygous Q175 mice (strain CHDI-81003003 (neo+), JAX Stock No: 027410) were intercrossed to produce homozygous (HOM), heterozygous (HET) Q175 mice and wild-type (WT) littermates (Charles River Laboratories, Sulzfeld, Germany). PCR confirmed the genotype of each mouse analyzed (Charles River Finland and Laragen Inc., Culver City, CA, USA). Mice were housed in groups of up to 5 per cage, in a temperature (22 ± 1°C) and humidity (30–70%) controlled environment with a normal light-dark cycle (7:00–20:00). The group sizes at different ages are described in Table 1. Both females and males were included, and all cohorts comprised a comparable (±1) number of animals of each genotype per respective age group (Table 1). There were no significant differences between sexes, and the data are shown for groups with animals of both sexes combined. All mice were housed in cages (dimensions: length 35 cm × width 19 cm × height 13 cm) with clean wood shavings covering the ground, changed weekly to provide the animals with dry bedding. A red mouse igloo was placed in each cage to provide environmental enrichment and shelter. Food (Purina Lab Diet 5001) and water were available *ad libitum* (Table 1).

**Table 1:** Number, age and sex of R6/2 and Q175 KI mice in experimental cohorts.

Cohort	Genotype	Age	Number of mice
R6/2	WT	4 weeks	16 F + 16 M
	TG	4 weeks	15 F + 17 M
	WT	10 weeks	11 F + 9 M
	TG	10 weeks	11 F + 10 M
Q175 KI HET	WT	3 months	10 F + 11 M
	HET	3 months	10 F + 11 M
	WT	6 months	10 F + 11 M
	HET	6 months	11 F + 11 M
	WT	10 months	6 F + 6 M
	HET	10 months	6 F + 6 M
Q175 KI HOM	WT	1 month	4 F + 5 M
	HOM	1 month	5 F + 5 M
	WT	3 months	4 F + 5 M
	HOM	3 months	5 F + 5 M
	WT	6 months	4 F + 5 M
	HOM	6 months	5 F + 5 M

WT, wild-type littermate; HET, heterozygous; HOM, homozygous; F, females; M, males.

### Fine motor skill and gait analysis

Fine motor skills and gait of the mice were captured by a high-speed camera (300 fps) under a brightly illuminated plexiglas corridor (153 × 5 × 10 cm, Motorater, TSE-systems GmbH, Bad Homburg, Germany). A few days before the test sessions, under light isoflurane anesthesia, the fur of the limbs was shaved. On the day of testing, the joints and tail of each mouse were highlighted with a non-toxic paint. The mice were analyzed while walking along the corridor and five or six complete strides were analyzed for each mouse. Only strides with continuous ambulatory movement were analyzed. Mirrors enabled the performance of the mouse to be detected simultaneously from left, right and ventral aspects. The movement of 24 points on each mouse were analyzed from the videos (Simi Reality Motion Systems, Unterschleissheim, Germany), and the trajectories of markers (see Table 2 for details) were analyzed by customized scripts.

**Table 2:** Anatomical landmarks marked and tracked in each mouse.

Term used in the text	Anatomical landmark^a^
Iliac crest	Iliac crest
Hip	Hip joint
Knee	Knee joint
Ankle	Ankle joint
Heel	Calcaneus
Paw	Distal metatarsal/carpal heads
Shoulder	Glenohumeral joint
Elbow	Cubital joint
Wrist	Radiocarpal joint
Nose	Nasus
Tail base	Tail base
Tail middle	Tail middle
Tail tip	Tail tip
Chin	Mentum
Front paw (from below)	Manus
Hind paw (from below)	Pes

^a^When applicable, the markers were applied to both left and right sides of the mouse. NA, not applicable.

Because the progression of behavioral changes in the transgenic mice was strain-specific, the time of each measurement was selected based on the results of standard behavioral tests of motor function in previous studies **[34–36]**. Specifically, we selected the initial ages of 4 weeks for R6/2 and Q175 HOM mice, and 3 months for Q175 HET, because those were the age points at which each model displays either mild or no motor deficits. We selected the older ages of 10 weeks for R6/2 mice, 3 and 6 months for Q175 HOM, and 6 and 10 months for Q175 HET animals because for each respective strain, there is documented temporal progression from mild to moderate and further, to severe stages of motor symptoms. Motor deficits are clearly apparent at 10 weeks in R6/2 mice, and at 6 months, they can be classified as advanced in Q175 HOM mice and as moderate in Q175 HET mice **[36]**. The data were combined from two or three experimental groups for each genotype, each experiment consisting of measurements at one or two age points from Q175 HET, Q175 HOM or R6/2 mice and the corresponding age-matched wild-type (WT) littermates.

### Measuring individual components of a complex spontaneous behavior

The components of spontaneous mouse motor activity were carefully defined. A full locomotor cycle, a stride, was defined as the period between two consecutive left hindlimb floor contacts. Parameter determination procedure was performed as follows: first, the initial ground contacts and the onsets of the swing phases for each limb were detected and strides were determined. Then, spatio-temporal parameters (e.g., stance and swing time, stride distance and mean speed), as well as parameters measuring inter-limb coordination, body posture and joint angles, and properties of limb trajectories during swing phase were determined. Altogether, 79 different parameters were established (Table 3).

**Table 3:** Definitions of kinematic parameters.

Parameter	Definition
**Spatio-temporal**	
Stride Time	Time to complete a stride
Mean Speed	Mean speed during ambulatory movement
Stride Distance	Distance moved during a stride
Stance Time (hind, fore)	Time that paw is in contact with the floor
Swing Time (hind, fore)	Time that paw spends in the air
Mean Swing Speed (hind, fore)	Mean speed of paw swing
Peak Swing Speed (hind, fore)	Maximum speed during paw swing
Swing Speed Metric (hind, fore)	Mean swing speed:peak swing speed ratio
Mean Swing Jerk (hind, fore)	Rate of acceleration change of a paw during middle half of swing
Swing Jerk Metric (hind, fore)	Mean swing jerk:peak swing speed ratio
**Inter limb coordination**	
Homolateral	Proportion of stride time when ipsilateral paws are both touching the ground or swinging
Homologous	Proportion of stride time when ipsi- and contralateral paws are both touching the ground or swinging
Diagonal	Proportion of stride distance in which a hindpaw and contralateral forepaw are both touching the ground or swinging
Left/Right Coupling (hind, fore)	Time difference between consecutive left and right ground contacts during a stride
L/R Coupling Deviation (hind, fore)	Deviation of left/right coupling between strides
Step Width (hind, fore)	The distance between forepaws or hindpaws when both are touching the ground during stance, perpendicular to midline
Step Width Deviation (hind, fore)	The deviation of step width between strides
**Posture**	
Toe Clearance (hind, fore)	Maximum distance of the paw from the ground during swing
Iliac Crest Height	Height of iliac crest during mid-stance
Mean Hip Height	Average height of the hip during a stride
Hip Height Range	Range of hip height (vertical movement) during a stride
Mean Hip Jerk	Rate of acceleration change of the hip during stride
Tail Base Height (min, mean, max)	Distance of tail base from the ground
Tail Base Height Range	Range of distances of tail base from the ground during a stride
Protraction (hind)	Maximum forward distance of the hindpaw with respect to the iliac crest during stride
Retraction (hind)	Maximum reverse distance of the hindpaw with respect to iliac crest during stride
Nose Height	Average distance of the nose from the ground during a stride
Nose Height Range	Range of distances of the nose from the ground during a stride
Lateral Head Rotation	Average degrees of the lateral head rotation during a stride
Head Rotation Deviation	Deviation of lateral head rotation between strides
Head Rotation Range	Range of degrees of the lateral head rotation during a stride
**Tail tip**	
Height (min, mean, max)	Distance of the tail tip from the ground during strides
Height Range	Range of tail tip heights during a stride
Tail Tip Over Hip	Percentage of stride time when the tail tip is higher than the hip
Ground Contact	Percentage of stride time when the tail tip touches the ground
Distance 2D	Ratio of the two-dimensional tail tip trajectory length to stride length, determined from the side view
Distance 3D	Ratio of the three-dimensional tail tip trajectory length to stride length
**Joint angles**	
Hip, knee and ankle angles (min, mean, max)	Angle of each joint during a stride
Hip, knee and ankle range of motion (ROM)	Difference between the maximal and minimal joint angles during a stride
Hip, knee and ankle ROM deviation	Deviation of joint ROM between strides
**Paw trajectory**	
Paw Trajectory Shape 25%, 50% or 75% (hind, fore)	Percentage of time the paw swings higher than 25%, 50% or 75% of the toe clearance
Toe Lift-Off Angle (fore, hind)	Angle of the paw ascent during an early swing
Relative Trajectory Length	(Forepaw 2D trajectory path length: stride length) ratio minus 1
Excess Vertical Movement	(Vertical forepaw trajectory distance: double of the toe clearance) ratio minus 1
Backward Paw Distance	Sum of excess backward movement of the forepaw during a stride

Strides were excluded from analysis if the animal stopped for any reason, or if the stride was unusually slow (slower than 50% of the median speed). The experimental setup measured both left, right and ventral aspects of each mouse simultaneously. All data are presented as an average of both left and right-side movements for each mouse strain.

### Data analysis

Principal component analysis (PCA) can measure the interdependence of kinematic parameters of the same movement and reduce the dimensionality of multivariate data set **[39–43]**. First, 26 principal components of z-score normalized kinematic gait parameter data were obtained, corresponding to 90% of total variance in the 63 kinematic parameters. After Varimax rotation **[44]**, five components (PC# 1, 2, 3, 5, and 11) were chosen based on the explained variance and the ability to differentiate the three genotypes from respective WT mice. The corresponding PC scores were obtained first for each stride of each individual, and finally, the mean PC scores were calculated over the strides.

To gain a comprehensive perspective on the spatio-temporal features of mouse gait, we established a method for marker trajectory data analysis and reconstruction to capture any correlated associations between different marker trajectories. The method is based on the PCA of an empirical data correlation matrix of marker trajectory data (also known as Karhunen-Loève transform) [**41**] and is independent of the subjectively pre-selected set of kinematic parameters. Similar approaches utilizing kinematic marker trajectory (waveform) data have been presented earlier [**39,45,46**]. In our approach, two-dimensional marker data of 22 selected markers of each stride (bilaterally: iliac crest, hip, knee, ankle, heel, hindpaw, shoulder, elbow, wrist and forepaw as well as single points of the chin and tail tip, middle, and base) were first referenced to the left iliac crest marker. The marker data were temporally normalized to 50 data points by interpolation, corresponding to 0–100% of the stride duration, adding up to 2,000 points per data vector. Empirical data correlation matrix (X^T^X/n) was formed from the data matrix X, consisting of data vectors from each stride of each mouse, and 50 PCs were computed. Finally, the PC scores were obtained and further incorporated into the phenotypic score.

### Fine motor phenotype scores

The phenotypic score was determined as follows: first, the average PC score of the WT group was subtracted from all PC scores. Second, the PC scores for each individual mouse were orthogonally projected onto a unit direction vector of the multidimensional line starting from the average of WT (zero after subtraction) and pointing towards R6/2 or Q175 groups within the PC space. Finally, the Phenotypic score was obtained based on the distances from WT. By definition, the average control group individual scored zero. The average disease model phenotype has a positive score. The magnitude of that value corresponds to the magnitude of phenotype-specific deviations in the overall gait pattern.

This approach used 63 separate parameters and trajectory waveform patterns to produce two separate overall phenotypic scores for each mouse. The unrotated eigenvectors were used to maximize the information available. The dimension of the PC score space was 26 and 50 for the parameter and marker trajectory data based on projected distances, respectively. Because there was also notable age progression in many parameters, the multidimensional lines connecting WT and TG group averages were determined separately for each age.

### Statistical analysis

Each of the 79 parameters was analyzed separately for every mouse strain at each age by using standard software (IBM SPSS Statistics 19 and StatsDirect). The comparisons of mutant genotype groups to the corresponding WT were performed by the unpaired *t*-test or the Mann-Whitney U-test. All values are presented as the mean ± standard error of the mean (SEM). Differences were considered significant when *p* < 0.05.

## Results

### Individual parameters of gait and fine motor features

Each mouse was assessed across 79 movement-related parameters per time point (Table 3). The overall timing and speed of motion were altered in R6/2 mice from 4 weeks of age. The stride time was longer, but the stride distance was shorter at 10 weeks, reflecting a longer stance time in both the hindlimbs and forelimbs compared to that in WT mice (*p* < 0.05, Table 4). In the forelimbs, the peak swing speed was slower at 4 weeks of age (*p* < 0.05, Table 4). In Q175 mice, the mean speed as well as the mean and peak swing speeds of the forepaws were slower from 6 months in the HET mice and from 3 months in the HOM mice (*p* < 0.05, Table 4). Furthermore, an ataxic movement at the peak of the swing phases was observed in the forepaws of R6/2 mice at 10 weeks of age. Representative example videos of R6/2 and corresponding WT mice at 10 weeks of age as well as of Q175 WT, HET and HOM mice at 6 months of age are included in Supporting Information (S1 Appendix).

**Table 4:** Summary of fine motor and gait deficits of R6/2 and Q175 mice during walking.

Kinematic parameter	Differences in kinematic parameters (%) between mutant and WT littermates over time								
	R6/2		Q175 KI						
			HET			HOM			
	MILD (4 weeks)	ADV (10 weeks)	MILD (3 months)	MOD (6 months)	ADV (10 months)	MILD (1 month)	MOD (3 months)	ADV (6 months)	
**Spatio-temporal**									
Stride Time		+24		+17	+34		+20	+26	
Mean Speed	−14	−41		−20	−25		−22	−30	
Stride Distance		−29		−6				−10	
Stance Time		H +52, F +52		H +27, F +26	H +60, F +50		H +31, F +31	H +41, F +38	
Swing Time				F +9	F +20			H +10, F +16	
Mean Swing Speed		H −21, F −28		H −9, F −13	F −17		H −15, F −15	H −14, F −21	
Peak Swing Speed	F −9	H −13, F −16		F −14	F −17		H −10, F −19	F −26	
Swing Speed Metric		H −8, F −12						H −11	F +6
Mean Swing Jerk		F +23		F −12	F −4			F −34	
Swing Jerk Metric		H +34, F +47							
Limb Coordination									
Homolateral IC		+58							
Homologous IC									
Diagonal IC		−16			−7			−7	
Left/Right Coupling									
L/R Coupling Deviation	H +35	H +81, F +46		H +29			H +60		
Step Width		H +22, F +30		H +8	H +7		H +12	F +20	
Step Width Deviation		H +52				H +50			
**Posture**									
Toe Clearance (Fore)	H −16, F −9	F −12						F +17	
Iliac Crest Height	-8	-7		−4	−12		−10	−18	
Hip Height	-13	-15		−11	−15		−23	−26	
Hip Height Range		+20					+28		
Mean Hip Jerk		+22		−12	+20				
Tail Base Height (Min, Mean and Max)	−32, −29, −26	−45, −37, −31		−22, −18, −16	−23, −19, −15		−41, −32, −26	−49, −44, −38	
Tail Base Height Range		+22				+16	+31		
Protraction (Hind)	+10	+25		+15	+25			+22	
Retraction (Hind)		−42							
Nose Height	−12	−23							
Nose Height Range			+16					+59	
Lateral Head Rotation				+36			+76		
Head Rotation Deviation							+53		
Head Rotation Range							+42		
** Tail Tip**									
Height (Min, Mean and Max)	−33, NS, NS	−57, −38, NS		−37, −30, −23			−54, −41, −30	−81, −62, −46	
Height Range					+34				
Over Hip				−32				−88	
Ground Contact		+49		+5.8 (absolute)					
Distance 2D		+15		+5	+8		+8	+8	
Distance 3D		+21		+7	+9		+11		
** Range of joint motion**									
Hip									
Knee	+7	+16							
Ankle					+24			+22	
Hip Deviation	+34	+58			+106		+61		
Knee Deviation		+48							
Ankle Deviation	+22			+37	+69			+50	
**Paw Trajectory**									
Paw Trajectory Shape 25%	F −9	H −6			H −12	H +4	H −6	H −18	
Paw Trajectory Shape 50%	H −9, F −16	H −11			H −22		H −17	H −36	
Paw Trajectory Shape 75%	H −19, F −18						H −18	H −40	
Toe Lift-Off Angle		F +28		H −19				H −24	
Relative Trajectory Length		+109		+26	+29		+60	+49	
Excess Vertical Movement	+34	+113							
Backward Paw Distance		+77		+34	+36				

(+ (highlighted in green), significantly increased compared to the values in the corresponding WT group; − (highlighted in red), significantly decreased compared to the values in the corresponding WT group; empty cell, no significant differences between the genotypes (*p* < 0.05, unpaired *t*-test/Kruskal-Wallis); WT; HET, heterozygous; HOM, homozygous; H, hindlimb; F, forelimb).

**Fig 1:**
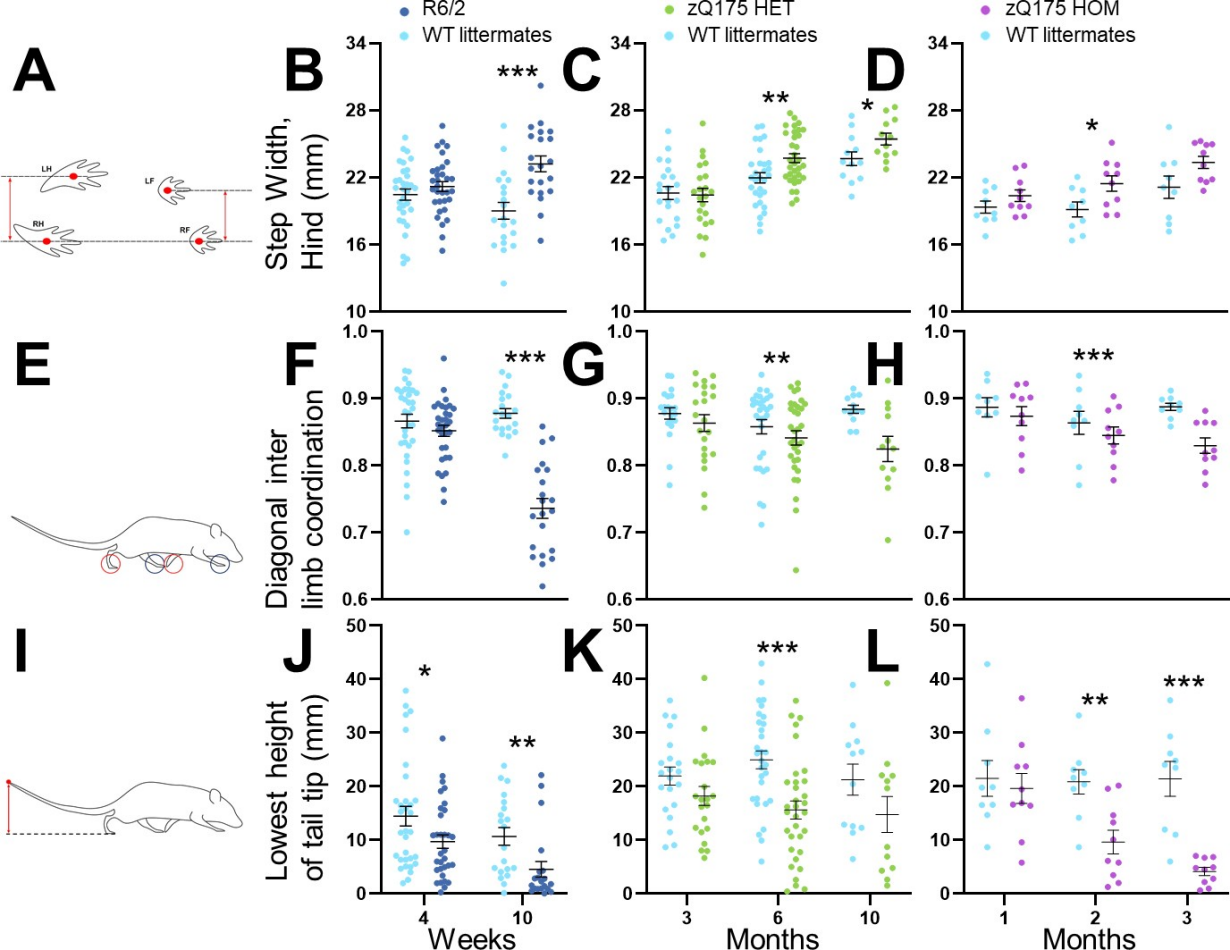
Gait phenotypes of R6/2 and Q175 mice during spontaneous motor activity. Histograms of the measurements from R6/2 (dark blue), Q175 heterozygous (HET, green), Q175 homozygous mice (HOM, purple) and wild-type (WT) littermates (turquoise) of step width (A–D), diagonal inter limb coordination (E–H) and height of tail tip (I–L) over time. Data are presented as the mean ± SEM. Statistical significance of the differences is indicated as follows: *p < 0.05, **p < 0.01, ***p < 0.001 (R6/2 / Q175 KI HET / HOM vs. WT, unpaired *t*-test). Information about the numbers of each group at specific ages is given in Table 1.

The cadence and interlimb coordination in R6/2 mice were significantly affected at 10 weeks of age. R6/2 mice took wider steps using all limbs, and the hindlimb step width was more variable (*p* ≤ 0.01; Table 4; Fig 1A and 1B). The diagonal cadence, the dominant form of cadence in normal healthy rodents, was substituted by the homolateral cadence (*p* < 0.05; Table 4; Fig 1E and 1F). Q175 mice also had deficits in the cadence and interlimb coordination but to a lesser extent than R6/2 mice. Specifically, hindlimb steps were wider in HET mice from 6 months and in HOM mice at 3 months (Fig 1C and 1D; *p* < 0.05). Forelimb steps were wider only in HOM mice at 6 months of age (*p* < 0.05, Table 4), with smaller diagonal cadence in HET mice at 10 months and in HOM mice at 6 months (*p* < 0.05; Fig 1G and 1H; Table 4).

Pelvic height was significantly lower in both R6/2 and Q175 KI mice. In the R6/2 mice, this was expressed, in particular, as decreased iliac crest height at mid-stance and mean hip height as well as increased protraction from 4 weeks of age (*p* < 0.05; Table 4). In the Q175 mice, iliac crest height and hip height were both decreased in HET mice from 6 months and in HOM mice from 3 months (*p* < 0.05; Table 4). Hindlimb protraction was increased from 6 months in both HET and HOM mice (*p* < 0.05; Table 4).

Tail tip position and movement of the tail were among the most robust motor changes in both R6/2 and Q175 mice. Tail tip height was lower at 10 weeks and the minimum height already from 4 weeks in R6/2 mice compared to the values in WT mice (*p* < 0.05; Fig 1I and 1J; Table 4). In addition, the tail tip spent longer on the ground, and the vertical and three-dimensional movement of the tail tip was increased during walking at 10 weeks (*p* < 0.05, Table 4). Tail tip heights were lower in HET mice at 6 months, and in HOM mice from 3 months (*p* < 0.05; Fig 1K and 1L). Similarly, the tail tips moved more in two and three dimensions at the same ages in both HET and HOM Q175 mice (*p* < 0.05; Table 4). The tail tip spent less time over the hip in both HET and HOM Q175 mice at 6 months (*p* < 0.05, Table 4).

In R6/2 mice, the range of motion in the knee was broader from 4 weeks to 10 weeks of age (*p* < 0.05; Table 4) and more variable in the hip (*p* < 0.05), knee (*p* < 0.05 at 10 weeks) and hind ankle (*p* < 0.05 at 4 weeks; Table 4). In the Q175 mice, the range of motion was broader in the ankle at 10 months in HET (*p* < 0.05) and at 6 months in HOM mice (*p* < 0.05). Further, it was more variable in the hip (*p* < 0.05) in HET mice at 10 months and in HOM mice at 3 months (*p* < 0.05), and for the ankle from 6 months in both HET and HOM mice (*p* ≤ 0.05; Table 4).

The trajectory shapes of front and hind paws during the swing phase of each stride, were significantly compromised in both R6/2 and Q175 mice. Lower trajectory profile of forepaws was observed in R6/2 mice at 4 weeks, in HET Q175 mice at 10 months and in HOM Q175 mice at 3 months of age (*p* < 0.05; Table 4). The major characteristics of the mouse lines—movement speed, interlimb coordination, pelvic height and paw trajectory shape—deteriorated in the groups of older mice (Table 4).

### Kinematic parameter PCA

**Fig 2:**
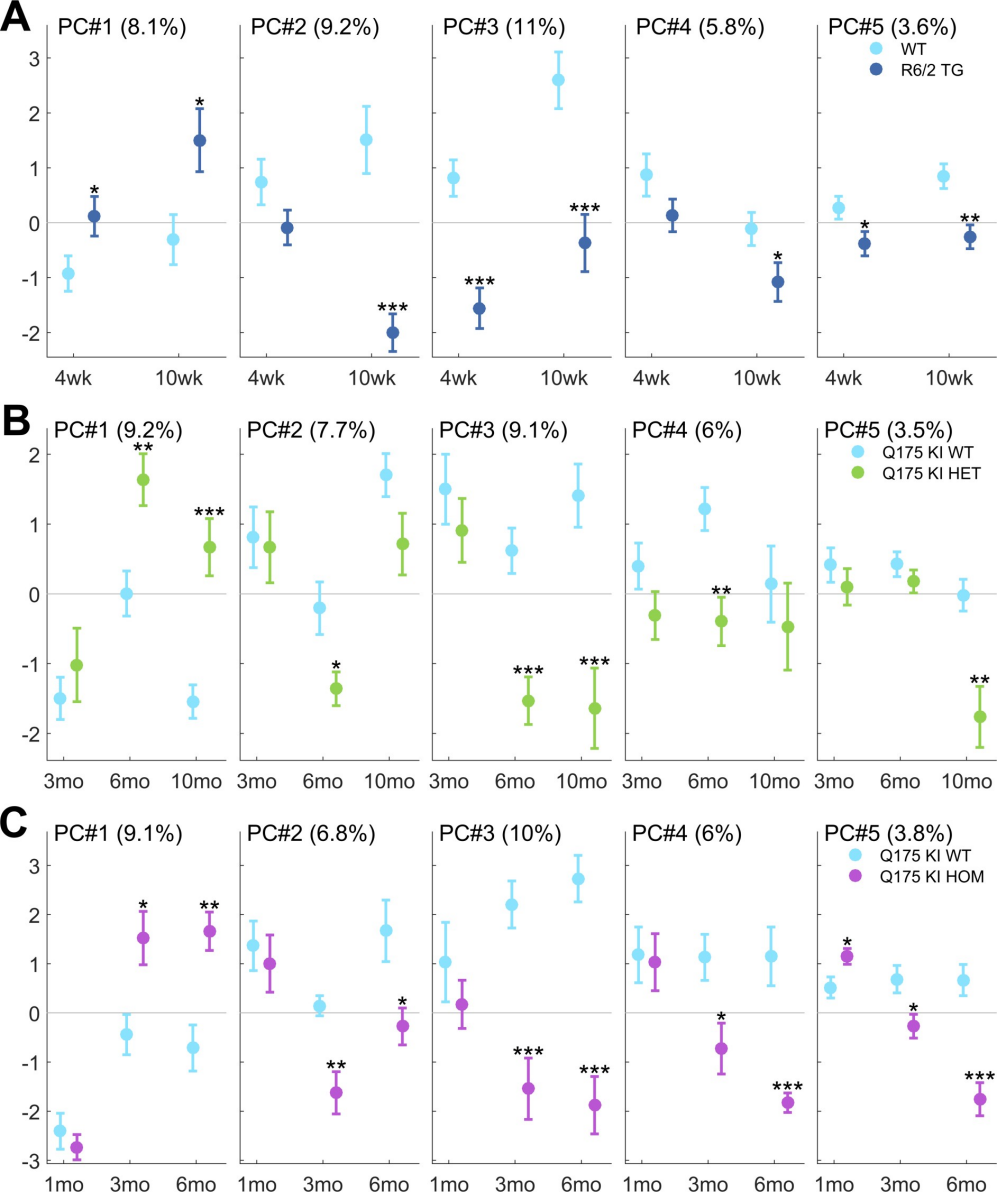
Fine motor and gait deficits using PCA. Principal component (PC) scores PC#1–5 of the five selected Varimax-rotated PCs are illustrated. The corresponding PCs (eigenvectors) are shown in Fig 3. Percentage in each panel describes the proportion of variation in the whole data set that each PC comprises. Data are shown separately for R6/2 (A), Q175 KI HET (B), and Q175 KI HOM mice (C). Data are presented as the mean ± SEM. Statistical significance of the differences is indicated as follows: *p < 0.05, **p < 0.01, ***p < 0.001 (R6/2 / Q175 KI HET / HOM vs. WT, unpaired *t*-test). Information about the numbers of each group at specific ages is given in Table 1.

**Fig 3:**
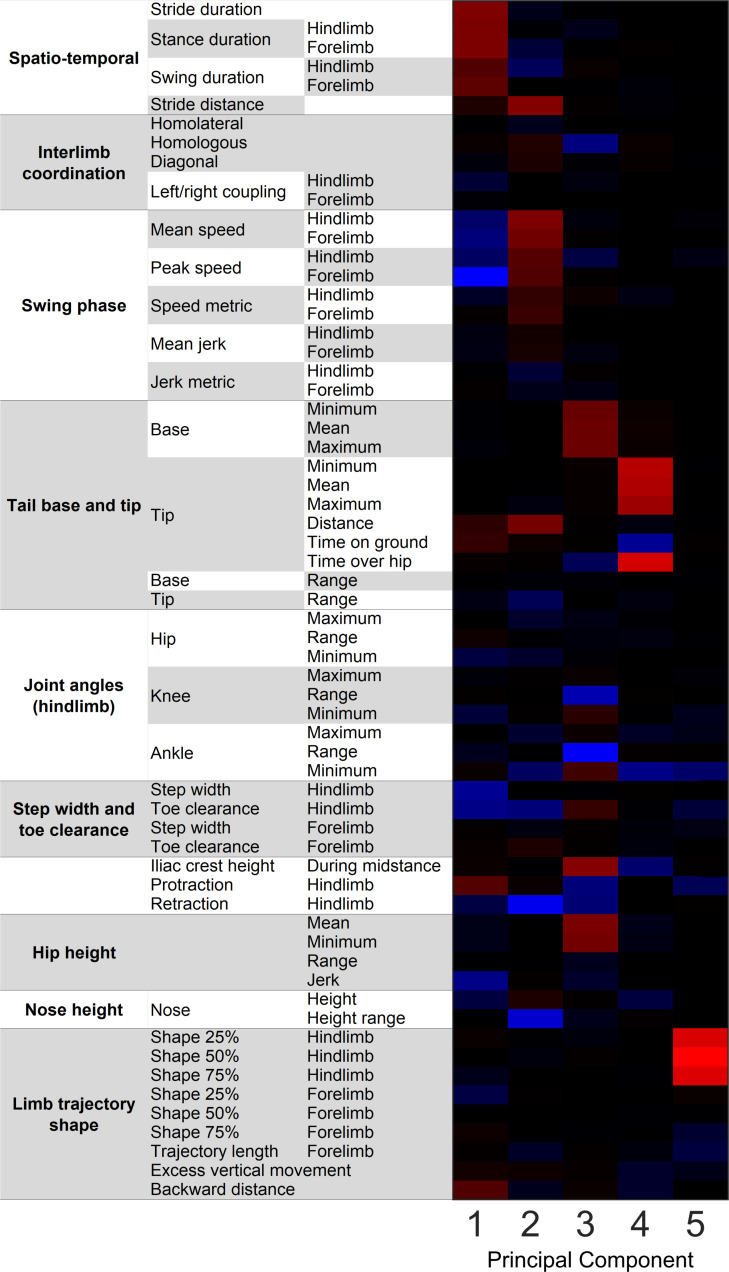
Principal components of gait phenotypes in R6/2 and Q175 mice. The five Varimax-rotated principal components are presented as a heat map, illustrating the interdependence of kinematic parameters within each component (red = positive correlation, blue = negative correlation, black = no correlation).

From the 63 interrelated movement parameters measured for each mouse, five principal components represented CAG repeat-associated pathological features: PC#1—overall slowness; PC#2—stride distance, swing speed; PC#3—pelvic height; PC#4—tail tip height; PC#5—swing trajectory profile (Figs 2 and 3). All transgenic mice moved slower than WT littermates (R6/2 from 4 weeks, Q175 HET from 6 months, and Q175 HOM from 3 months; *p* < 0.05 for PC#1 in all groups). R6/2 mice at 10 weeks, Q175 HET mice at 6 months, and Q175 HOM mice from 3 months old had slower swing speeds and shorter strides than WT mice (*p* < 0.05 for PC#2 in all groups). Pelvic height was the component that most consistently differentiated HD mice from their WT littermates. Both R6/2 and older Q175 mice walked with their body closer to the ground than WT littermates (*p* < 0.05 for PC#3 in all groups), although for the youngest Q175 age groups (3-month-old HET mice and 1-month-old HOM mice), no difference was observed. In addition to hip, iliac crest and tail base height parameters, the body posture PC#3 also represented inversely correlated knee and ankle ranges of movements and pro/retractions, meaning that a reduced PC#3 indicated both lower body posture and increased vertical movement, or worse body control.

PC#4 represented the height of the tail tip, which was significantly lower in R6/2 at 10 weeks (PCA; *p* < 0.05), in Q175 HET at 6 months (*p* < 0.05), and most distinctly in Q175 HOM from 3 months (*p* < 0.05). Overall, the tail tips of HD mice were closer to the ground than those of WT animals.

PC#5 represented the limb swing trajectory profile. HD mutants demonstrated lower hind limb trajectories and greater protraction. The maximal toe clearance was not different between HD and WT mice, but the paw trajectory shape was different in the mutants, namely, the hindpaw was closer to the ground during the swing phase. This principal component showed deficits in R6/2 mice at all ages (*p* < 0.05), in Q175 HET mice at 10 months (*p* < 0.05) and in Q175 HOM mice from 3 months (*p* < 0.05).

**Fig 4:**
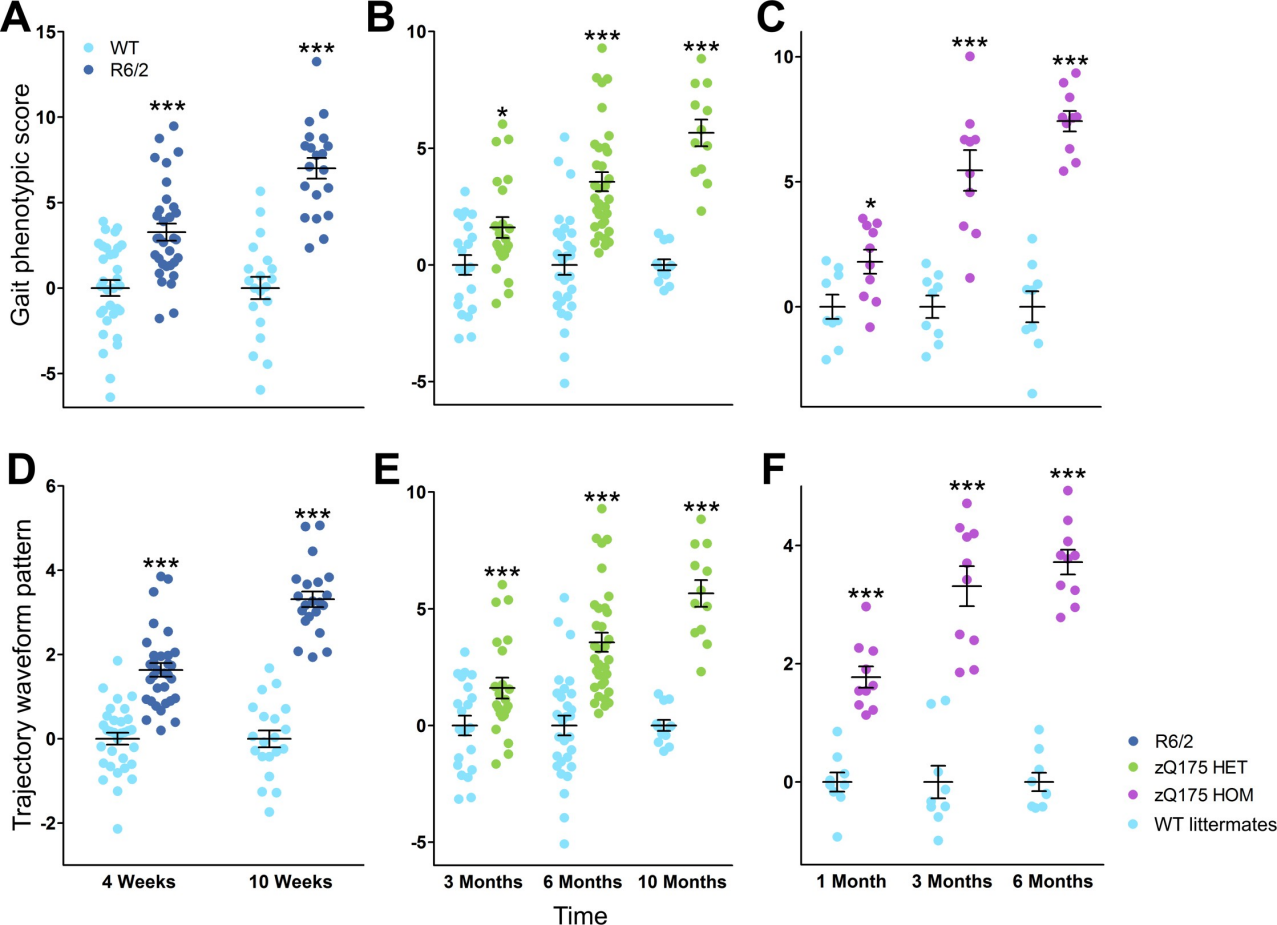
Gait and trajectory data phenotypic scores in R6/2 and Q175 mice worsen over time. Graphs of the scores from each R6/2 (dark blue), Q175 heterozygous (green), Q175 homozygous mice (purple) and wild-type (WT) littermates (turquoise) after PCA for either 63 gait parameters (A–C) or 24 parameters of marker trajectory data (D–F) over time. Data are presented as the mean ± SEM. Statistical significance of the differences is indicated as follows: *p < 0.05, ***p < 0.001 (R6/2 / Q175 KI HET / HOM vs. WT, unpaired t-test). Information about the numbers of each group at specific ages is given in Table 1.

Finally, two Fine Motor Phenotype Scores were calculated by combining the kinematic parameters (Fig 4A–4C) and the interpolated marker trajectory data (Fig 4D–4F). R6/2 mice demonstrated altered gait parameter and marker trajectory data-based scores at both 4 and 10 weeks of age (*t*-test; *p* < 0.05) (Fig 4A and 4D). Similarly, Q175 HET and HOM mice had altered gait and marker trajectory scores from as early as 3 months and 1 month of age, respectively (*t*-test; *p* < 0.05 for all comparisons) (Fig 4B, 4C, 4E and 4F). Importantly, no single parameter was as sensitive as the Fine Motor Phenotype Scores for detecting early phenotypes in the movement of the mice: whereas no single parameter allowed for reliable differentiation of *all* age groups in the three cohorts (Table 4), both phenotypic scores were significantly different for mutants of all ages compared to the values in respective WT counterparts (Fig 4). Furthermore, power analyses demonstrated that the Fine Motor Phenotype Score based on the marker trajectory data required fewer mice to detect a prospective 50% treatment effect in young mice that the score based derived from the kinematic parameters (Table 5).

**Table 5:** Power analysis for the fine motor phenotype scores computed from kinematic parameter and marker trajectory data.

Mouse strain	Age	Predicted group size to show 50% treatment effect	
		Kinematic Parameters	Marker Trajectories
Q175 HET	3 months	101	17
	6 months	29	11
	10 months	6	8
Q175 HOM	1 month	45	7
	3 months	11	7
	6 months	5	3
R6/2	4 weeks	45	19
	10 weeks	12	6

Calculated group size *N* required for the detection of 50% recovery (that is, 50% of the effect size between WT and TG). Alpha = 0.05; power = 0.8; effect size: Hedges' g.

## Discussion

Gait disturbances are important manifestations of HD, therefore gait features are typically assessed in standardized HD motor scores, such as UHDRS-TMS, which are frequently used in HD clinical trials **[47]**. Although various motor assessments are a conventional element of mouse HD model phenotyping, specific analysis of gait is seldom carried out. Here, to provide an integrative tool for drug discovery, we measured 79 locomotor parameters in each individual mouse to comprehensively characterize motor phenotypes in two genetically engineered HD mouse models during disease progression. From these individual constituents of mouse locomotion, we identified five central themes (principal components) and derived two functional phenotypic scores based on kinematic and marker trajectory indices that robustly quantified locomotor deficits. We showed that our Fine Motor Phenotype Scores more sensitively detected changes in locomotion in mutants than any single kinematic parameter and therefore, these scores may be applied for characterization of other HD mouse lines, as well as mouse models of other diseases associated with locomotor impairments.

Motor dysfunction in HD mouse models has been commonly detected by the open field and rotarod tests as part of preclinical evaluations of numerous potential HD therapeutics **[48,49]**. In addition, the utility of specific gait measurements in HD mouse models is becoming increasingly acknowledged, because gait alterations in HD patients may have different sensitivity to the available treatments compared to other motor manifestations, such as chorea **[50,51]**. Treatment-induced improvements of gait abnormalities in HD mouse models have been described in several published reports. We have recently observed a positive effect of intracerebroventricular infusions of an antisense oligonucleotide against the expanded CAG repeat in HTT mRNA on stride distance, nose height and peak swing speed of R6/2 mice **[13]**. Intrastriatal injections of a calmodulin fragment and dantrolene administration with food significantly increased the reduced stride length in R6/2 mice **[52]** and YAC128 mice **[33]**, respectively. Intracerebroventricular infusion of ganglioside GM1 shortened stride duration, corrected stance‐to‐stride ratio and restored interlimb coupling in Q140 mice **[53]**. Notably, transplantation of embryonic stem cell-derived neural progenitors into the N171-82Q mouse model of HD rescued changes in stride length, print length and print area as revealed by the CatWalk gait assay, whereas conventional grip strength and rotarod tests failed to demonstrate positive effect of the treatment **[54]**.

Progressive decreases in movement speed and lower stride distance observed by us in R6/2 and Q175 mice (Table 4) are in agreement with previous assessments of locomotor functions in these **[13,37,38,54–58]** and other **[29–31,33]** mouse models of HD. Here, we replicated our previous findings of lower stride speed, stride distance and peak swing speed in 10-week-old R6/2 mice **[13]** and extended some of them to the younger age of 4 weeks. Shorter stride distance was also noted in 10-week-old R6/2 mice in the CatWalk **[54]**. In contrast, experiments in the Digigait automated treadmill video capture system failed to reveal differences in stride distance, stride time or swing time in 17-week-old R6/2 mice and their WT counterparts although expected shorter stride distance was revealed in R6/2 mice in that study by using conventional footprint test **[38]**. Moreover, the stance time was shorter in the mutants in that study, whereas in our experiments, it was longer. These discrepancies are likely explained by the forced nature of treadmill testing and differential adaptation to it by WT mice and mutants at the advanced stage of the pathology **[38]**.

In addition to comparing individual parameters, we also calculated two Fine Motor Phenotype Scores based, respectively, on the kinematic indices (Fig 4A–4C) and interpolated marker trajectory data (Fig 4D–4F). Similar approaches have measured patterns of gait changes in patients: scores such as the Gillette Gait Index and Gait Deviation Index are based on the principal components of pathological gait or kinematic waveform data, respectively, without predetermined parameters, and they can be used to assess treatment efficacy **[3,39,59]**. Recently, a computational approach that utilized support vector machines was applied to a large-scale analysis of a series of HD KI mouse line, including HET Q175 animals, to reveal behavioral features that would allow clear differentiation of mice with CAG repeats of different lengths **[60]**. Multiple spatio-temporal gait features of the KI lines were determined in the NeuroCube apparatus, where animals walked for 5 min, and the change in base width was among top 10 parameters that allowed to reliably differentiate six KI mouse lines with the number of CAG repeats ranging from 20 to 175 **[60]**. As detailed descriptions of the individual parameters and their changes were not explicitly reported in that study **[60]**, it is impossible to compare adequately the sensitivity of the NeuroCube and our Motorater-based approaches to detect gait changes in Q175 mice. A potential advantage of the NeuroCube-based method is that it integrates more parameters over longer period of spontaneous walking. On the other hand, as it is not clear whether and how different anatomical parts were marked in that study, it is likely that our approach allowed better estimation of the movement trajectories.

In our present experiments, the strongest principal component of movement in both HD models was the slowing of the time between strides, consistent with similar findings in rodent models of other neurodegenerative diseases **[61–65]**, and people with either Huntington’s or Parkinson’s diseases **[66]**. Our approach allows us to measure the interactions between slower movements and other gait parameters, especially those related to stride tempo. Slower movement correlated with lowered hind limb paws and shorter steps, consistent with the data in the Hdh^(CAG)150^ KI HD mouse model **[32]**. Walking speed is linked to limb coordination **[67]**, and the limbs of healthy mice are remarkably coordinated during walking **[68]**. Our data were consistent with evidence from mouse models of HD **[54]** as well as neurological disorders and traumatic brain injury **[69–75]** in revealing decreased limb coordination in the mutants. In contrast, parameters related to the body posture–for example, the range of joint movement–were uncoupled from walking speed in our experiments, consistent with observations of R6/2 mice in a forced walking test **[76]** or during spontaneous movement **[37]**. In addition, the emergence of forelimb ataxia in the R6/2 mice is in accord with case reports of ataxia symptoms in HD patients **[77,78]**, although mouse models of spinocerebellar ataxia exhibit different kinematic patterns **[79,80]**. Another important characteristic in both R6/2 and Q175 mice was the lowering of the rear body. The lower base of the tail was related to several other body posture parameters, including lower iliac crest, hip and hind limb paw clearance, and changes in the range of joint movements. Decreased height of the iliac crest was also reported in male (but not female) Q140 mice **[54]**. These data suggest postural instability in the mutant mice, consistent with clinical examinations of HD patients, for whom falls are a common problem **[81,82]**. Further studies are required to determine whether these movement phenotypes are primarily due to altered brain circuits, motor neuron dysfunction or whole-body changes in metabolism. The body weight of both R6/2 and Q175 mice decreased over time, but we did not include this factor in our analyses: in Q175 mice, this weight loss began after the observed motor deficits, indicating that it was unlikely a direct cause of the observed locomotor changes **[36,37]**.

The earliest changes in the whole brain, cortical or striatal volumes are detected at 2, 3 and 4 months of age in R6/2, Q175 HOM and Q175 HET mice, respectively **[36,83]**. That fact that our phenotypic scores allowed distinguishing significant phenotypes in the same models at 1, 1 and 3 month, respectively (Fig 4), indicates that the approach suggested by us can detect changes between mutant and WT mice even before neuronal loss starts. Power analysis revealed that the phenotypic score based on marker trajectories was more sensitive than that based on gait parameters in differentiating the transgenic mice from their WT littermates, indicating that the trajectory waveform patterns are more useful, albeit harder to interpret, than a set of parameters derived from the same raw data. Moreover, the trajectory data-based method can be considered more objective, because the parameters used were not subjectively selected. However, the kinematic parameter-based approach may be easier to interpret, as the individual parameters can be traced and emphasized in the final score. Another limitation in the trajectory data-based phenotype score is the loss of absolute speed information, because the method is based on interpolated trajectory data.

In summary, our hypothesis-free analysis of mouse movements both improved the statistical power of motor function assessment for therapeutic studies and detected early gait changes more sensitively than the conventional motor tests. The behavioral test described here uses data obtained during spontaneous locomotion and requires neither extensive training nor pharmacological induction. We show that robust phenotypes in mice can be observed early, within 1 month of age for R6/2 mice, shortening *in vivo* studies and potentially accelerating drug discovery. The data capture and analysis are semi-automated, maximizing study objectivity while minimizing chances for data handling mistakes. In addition, this behavioral test takes less than 20 seconds per mouse, enabling multiple cohorts to be tested by the same handler on the same day on the same apparatus, minimizing potential confounding effects **[84]**. Data analysis and generation of phenotypic scores is rapid, and interpretation of phenotypic changes is easy. Overall, our method improves the translational toolbox to evaluate HD therapeutics and is applicable to other movement disorders.

## Supporting information

S1 Appendix (ZIP)

S1 Graphical Abstract (TIF)
