# Supplementary figures and images for: Rapid and robust patterns of spontaneous locomotor deficits in mouse models of Huntington’s disease
Source: PLoS One. 2020 Dec 28;15(12):e0243052. doi: 10.1371/journal.pone.0243052 (PMC7769440; doi:10.1371/journal.pone.0243052)

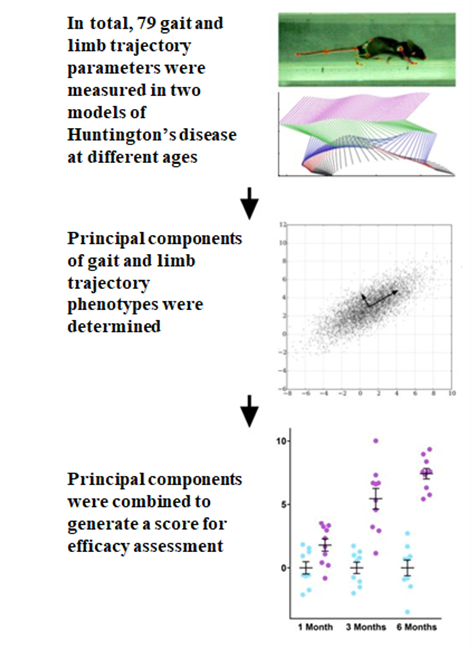

Supplement: S1 Graphical Abstract — (TIF) [file pone.0243052.s002.tif]
